# Political vs. expert: the impact of influencer type on vaccination willingness

**DOI:** 10.3389/fpubh.2026.1735159

**Published:** 2026-04-10

**Authors:** Jiangyi He, Jinyan Hu, Yiyang Li, Yanfei Luo

**Affiliations:** 1 School of Journalism and Communication, Henan University, Kaifeng, China; 2 School of Creative Media, City University of Hong Kong, Hong Kong, Hong Kong SAR, China; 3 School of Journalism and Communication, Zhengzhou University, Zhengzhou, China

**Keywords:** influencer type, message framing, pandemic, perceived credibility, vaccination

## Abstract

**Introduction:**

During a pandemic, influencers played a key role in promoting vaccination. This study examines the impact of influencer types (political vs. expert) on vaccination in the context of a future pandemic caused by a novel virus.

**Methods:**

A 2 × 2 between-subjects experimental design was employed, with 287 valid adult participants recruited from China. Among all participants, 41.81% were male and 58.19% were female, with an average age of 30.20 years (SD = 7.64). Participants were exposed to simulated social media posts that manipulated influencer types (political vs. expert) and message framing (gain vs. loss). Influencers were presented with different profile pictures, nicknames and background descriptions, while follower counts remained unchanged. Message framing was manipulated by presenting vaccination information using a gain frame (emphasizing the benefits of vaccination) or a loss frame (emphasizing the risks of non-vaccination). Analysis of variance (ANOVA) was used to test the impact of influencer type, and its interaction with message framing on vaccination intentions. Meanwhile, the mediating effect of perceived credibility was tested using the PROCESS Macro Model 4 in R.

**Results:**

Results indicate a key main effect of influencer type on vaccination willingness (*p* < 0.01, *η*_p_^2^ = 0.04). Political influencers (M = 5.93, SD = 0.93) were more persuasive than expert influencers (M = 5.60, SD = 0.81) in promoting vaccination. This effect was mediated by perceived credibility (*b* = 0.17, boot CI = [0.003, 0.332]). A significant interaction existed between influencer type and message framing (*p* < 0.05, *η*_p_^2^ = 0.015).

**Discussions:**

Our findings highlight the importance of political influencers during pandemics, a discovery consistent with the informational entropy-based notion of value. This persuasive effect is achieved by enhancing perceived credibility, which can be explained by meaning transfer. Political influencers often align with mainstream authorities, and audiences view their messages as the government’s stance. Therefore, the public’s trust in the government shifts to political influencers, thus enhancing their influence during the pandemic. Interaction analysis indicates that correctly matching influencer type and message framing enhances vaccination intention. This matching effect is driven by the image the influencer represent. These findings contribute to more effective vaccine promotion during pandemics.

## 1 Introduction

The COVID-19 pandemic has profoundly impacted global public health and economic development, and the risk of future pandemics caused by unknown viruses remains. Persuading the public to get vaccinated to achieve herd immunity will be key to ending such unknown pandemics. However, the presence of vaccine hesitancy poses a significant challenge to persuading the public to get vaccinated during pandemics. Therefore, how to reduce vaccine hesitancy and increase people’s willingness to get vaccinated through communication strategies remains a major public health issue during pandemics. Although the content of information (such as the gain and loss frames) plays a significant role in influencing vaccination decisions, an increasing amount of evidence indicates that the type of information source is even more important. During information processing, the type of information source serves as a key cue in heuristic processing, while the content of information functions as a central cue for systematic processing. During the pandemic, due to limited knowledge about the virus and information overload, the public has become more inclined toward heuristic processing patterns while reducing systematic elaboration (1, 2). Therefore, the type of information source matters more than message content alone.

The source of a message is an important factor that shapes public health attitudes and behaviors (3). For this reason, influencer-based communication strategies in social marketing are often used in vaccination campaigns to address vaccine hesitancy. For example, public figures, specially experts and officials, frequently use social media to persuade the public. This has made influencer effectiveness an important issue in health communication research. Although prior studies have paid considerable attention to the role of influencers in vaccine promotion, their conclusions remain divided. In particular, evidence regarding the effectiveness of expert sources is mixed: some studies report positive effects, whereas others find no significant influence (4). On the other hand, information fatigue has led some social media users to grow weary of and more skeptical about expert voices. For example, the phrase “*It is suggested that experts stop making suggestion*s” once became a trending topic on Weibo, a major social media platform in China (5). Although prior studies have found that vaccine-related posts by politicians receive lower levels of social endorsement (e.g., likes) than those posted by experts (6), this does not directly indicate that political influencers are less effective at persuading the public to get vaccinated. More importantly, existing literature lacks systematic comparisons and causal tests of the differences in persuasive effectiveness between political and expert influencers in vaccine promotion. Therefore, it is crucial to systematically examine the different effects of political and expert influencers in vaccine promotion. This kind of research can provide scientific guidance for improving future vaccination campaigns. In this study, political influencers refer to content creators who post content on social media in support of political positions or social causes (7); expert influencers refer to those who have established a professional reputation in a specific field (8), and who influence audiences’ views, behaviors, and other responses.

Source credibility models hold that the positive traits of communicators may have an impact on individuals (9, 10). In vaccine communication, the public usually associate political influencers with institutional authorities and policy communicators, while connecting expert influencers with expertise and competence. These distinct cues may affect people’s interpretation of vaccine-related information, which in turn affects their behavioral intentions. This provides a theoretical basis for exploring whether there are differences in the willingness of different influencer types to get vaccinated. Moreover, source credibility is not merely an outcome but also a psychological mechanism for influencers to exert persuasiveness. Accordingly, we hypothesize that perceived credibility mediates the relationship between influencer types and vaccination intention.

Framework theory suggests that health information can be presented in terms of gains (emphasizing the benefits of vaccination) or losses (emphasizing the risks of not getting vaccinated) (11). In health communication, message framing strategies have been widely used to promote preventive health behaviors, including vaccination. However, the results of previous studies on the persuasive effects of message framing have yielded inconsistent findings [e.g., (12–14)]. This indicates that the framing effect may depend on other situational factors. The heuristic-systematic model holds that individuals process persuasive information through both heuristic and systematic processing, which can coexist and interact with each other (15). This suggests that there may be an interaction between influencer type acting as a heuristic cue and message framing acting as a systematic cue. Therefore, this study further proposes a research hypothesis that influencer type interacts with message framing in influencing vaccination intention. Examining this interaction can advance theoretical understanding of how information source and message content jointly shape persuasion, and also has practical implications for designing more effective vaccine communication strategies.

Based on this, the present study simulated a pandemic caused by a hypothetical X virus. Against this backdrop, a 2 (influencer types: political vs. expert) × 2 (message framing: gain-framed vs. loss-framed) between-subjects experiment was conducted to examine the effect of influencer type on vaccination intention, as well as to test the interaction between influencer type and message framing. The findings of this study are expected to help promote vaccination during pandemics, enhance public willingness to get vaccinated, and contribute to ending pandemics as early as possible.

## 2 Literature review and hypothesis

### 2.1 Political influencers and expert influencers

Influencers are individuals who possess a strong social network and can exert significant influence over their followers (16–18). The rise of social media platforms (such as X, YouTube, Facebook, Weibo, Xiaohongshu, and others) has provided influencers with online communication channels, leading to the emergence of social media influencers. As a new type of celebrity, they are typically active on one or more social media platforms, building a large following by consistently posting content that appeals to their target audience (19), and establishing themselves as credible leaders in one or more specific domains (16). In the age of social media, influencers are an important source of information. They are not only providers of information, but also vital factors in the persuasion process (3). With their personal image, fan base, and content influence, influencers have become very persuasive sources, which has a significant impact on the public’s attitude and behaviors (20). During the pandemic, they can even influence health habits (21), such as getting vaccinated, wearing masks, keeping social distance, staying home, and washing hands.

In persuasion research, many scholars classify influencers according to the number of followers [e.g., (22, 23)]. However, this approach overlooks the individual differences among influencers (24), such as their background characteristics and expertise. In light of this and considering the practical implementation of vaccine promotion campaigns, our study divides influencers into two groups: political influencers and expert influencers.

Political influencers refer to content creators who support specific political positions, social causes or candidates by creating or sharing media content on specific social media platforms (7). This group often includes political leaders, presidential candidates, and senators. They typically use government resources or policy discussions to spread their message and play an important role in public issues. Expert influencers refer to the group that builds a professional reputation by relying on professional knowledge, skills or experience in a certain field (8), and then influences the opinions, behaviors, and decisions of their followers. Expert influencers are people from many different fields or professions.

Political influencers and expert influencers are playing important roles in vaccine promotion. In detail, political influencers can resonate with followers (25), and they are effective with groups that have high levels of political trust. This may be because political influencers are usually closely related to state authority and government discourse. They are symbols of institutional authority, conveyors of political positions, or transmitters of policy signals. Through this symbolic association, they can influence attitudes and behaviors by shaping identity and strengthening group norms (26), which arouse the public’s emotional resonance and value orientation on social media. In contrast, as a reliable source of vaccine information, expert influencers (e.g., healthcare professionals) can influence their vaccination decisions by raising public awareness (27, 28). This is because expert influencers are typically perceived as symbols of rationality and objectivity, and their persuasive power primarily stems from public recognition of their expertise (29), rather than from emotional resonance.

However, there is still a key issue pending in vaccine campaigns. We still do not know which type of influencer, political or expert, is more persuasive as a source of information. In relevant research, the role of experts as important sources of information has received extensive academic attention. Although some studies have found that experts have a positive impact on vaccine promotion, others have come up with different results (4). Other studies have shown that vaccine-related posts published by experts have received more likes than those posted by politicians (6). However, that does not mean that experts are necessarily more effective than politicians in persuading people to get vaccinated. The COVID-19 pandemic has made this problem even more complex. During the crisis, public skepticism about these two types of influencers grew, leading to uncertainty about their effectiveness. For example, political leaders were often questioned by the public for making statements that seriously underestimated or misrepresented the severity of the epidemic. A survey report from 2022 showed that public confidence in scientists and medical experts had declined sharply, even below the pre-epidemic level (30). More importantly, there is a serious lack of prospective experimental studies, which leads to a significant gap in understanding the differences in the persuasive effects of political influencers and expert influencers on vaccination promotion.

Theoretically, the existing literature provides limited guidance on potential differences between political influencers and expert influencers in terms of persuasive impact. In practice, both types of influencers may face public skepticism. Given these theoretical and practical uncertainties, this study adopts an exploratory approach to examine the differences in persuasive effects between political influencers and expert influencers.

Accordingly, the following research question is proposed in this study.

*RQ1*: In the promotion of vaccines, does the effectiveness of encouraging public vaccination differ between political and expert influencers?

### 2.2 Mediating effect of perceived credibility

Perceived credibility refers to the receiver’s overall perception of the credibility of the information being conveyed (31). Many studies show that credibility is a key factor influencing persuasion effectiveness in health communication [e.g., (32, 33)]. When promoting vaccines, the public’s perceived credibility of information from different influencers in different ways. Then this can affect their decision to get vaccinated.

Source credibility models focus on the positive traits of communicators. These traits decide if people would accept a message (10, 34). People usually trust experts because of their “expertise” and “competence” (35). Specifically, experts have rich knowledge in the fields of health and medicine. This enables them to provide professional advice and thus win the trust of the public (6). Research shows that during the pandemic, politicians had more influence than celebrities (36). This is because political influencers, lack professional knowledge, can win public trust with their inherent authority and ability to resonate emotionally with the public. Although it is not clear which type of influencer, political or expert, will be perceived as more credible during future pandemics, existing research suggests that the perceived credibility of messages will vary depending on the source [e.g., (32, 37)].

Previous studies have shown that credibility is a key factor in public health choices. For example, Borah (38) shows that perceived credibility has a significant impact on vaccination willingness. Furthermore, under conditions of low elaboration likelihood, source credibility mainly influences individuals’ information processing through the peripheral route (31, 39). This often happens in vaccine promotion campaigns, as people may have limited health literacy or feel overwhelmed by information. In such cases, people do not usually analyze messages in detail. Instead, they rely on simple cues to make decisions. When a source appears trustworthy, audiences are more willing to accept the information it provides. This reliance on credibility reflects the use of the peripheral route in message processing. As a result, messages delivered by credible sources have a greater chance of shaping attitudes. In the context of vaccines, credibility can increase the likelihood that individuals will accept external information. Over time, this acceptance can strengthen their intentions to get vaccinated. In addition, the Bayesian Mindsponge Framework (40, 41) provides a new theoretical perspective for explaining this behavior of the public. As an information-processing framework, the core of this theory, the mindsponge mechanism (42–44) regards the human mind as a dynamic information processing system (45, 46). The system uses a benchmarking process to decide whether to absorb or reject new information. During this process, as the information filtering process can be time-consuming and energy-consuming, the human brain may treat “trust” in the information source as the gatekeeper of priority information channels (47, 48). In other words, the presence of trust affects how much information a person absorbs, and this then influences their subsequent behavior (49, 50).

Building on this, the following hypothesis is proposed:

*H1*: Perceived credibility mediates the relationship between influencer types (political vs. expert) and vaccination intention.

### 2.3 Interactive effects of influencer type and message framing

Besides the information source, in health communication, message framing is regarded as a common strategy for promoting behavioral change (4). There are two main types of frames: a gain frame highlights the benefits of doing something, while a loss frame highlights the costs of not doing it (51, 52). For instance, in a vaccine promotion campaign, a gain frame message might say “*Get vaccinated can prevent with the virus*,” while a loss frame message might state “*Not getting vaccinated may put you at risk of infection*.” Research on message framing indicates that the persuasive effects of framing depend on the type of behavior (53). The gain frame is suitable for encouraging behaviors with lower risks, such as preventive behaviors; while the loss frame is more likely to encourage behaviors that imply greater risks, such as detection behaviors (54, 55). Given that vaccination is considered a preventive behavior, gain-framed messages are more likely to promote public vaccination. This assertion is supported by empirical findings in previous research [e.g., (14, 56, 57)]. However, some studies have reached inconsistent conclusion. For instance, some studies found that there is no significant difference between gain-framed messages and loss-framed messages for promoting vaccination (12, 58–60). In contrast, other studies have concluded that loss-framed messages were actually more effective (13, 61, 62). These contradictions suggest that the effectiveness of message framing is conditional (55). In other words, there may be an interaction effect between message framing and other factors.

As an important theory in the field of persuasive communication, the heuristic-systematic model (HSM) holds that the heuristic processing and systematic processing can coexist and interact with each other (15). In the process of processing information, the source features and content features, respectively, correspond to these two processing methods (33). When persuading individuals to get vaccinated, the influencer type pertains to the “source features” of the information, serving as a key cue for heuristic processing, whereas message framing pertains to the “content features,” functioning as an important basis for systematic processing. In addition, existing research has examined the interaction between message sources and message framing, and has found that when specific sources are combined with specific message framing, the persuasive effect of the information is enhanced (63). This indicates that there may also be a significant interaction between influencer type and message framing, playing a key role in changing behavioral intentions.

Based on this, the next hypothesis is proposed:

*H2*: Influencer type interacts with message framing in influencing vaccination intention.

## 3 Method

### 3.1 Study design

All participants were randomly assigned to one of four experimental conditions (2 influencer types × 2 message framing). Randomization was automatically performed by the survey platform through its built-in random assignment feature to ensure similar distributions of characteristics across experimental groups. We introduced the background of the experiment to all participants and asked them to imagine the following scenario: The world faced a pandemic caused by an unknown pathogen. The World Health Organization named this disease “Virus X.” Like COVID-19, the virus can be transmitted through the respiratory tract, with a high infection rate and mortality rate, and it is difficult to control. Due to the lack of effective treatments, vaccination to acquire antibodies became an important measure to prevent infection. Afterwards, we presented the experimental scenario to the participants and asked them to read a post published by a political influencer or an expert influencer from Weibo. We chose Weibo for the following reasons: like Twitter, it is an open platform; it has a large user base and is one of the most popular social media platforms in China; and people in China frequently use it to share or search for health-related information. At the beginning of the post, the following information was presented to participants: “The X vaccine is a vaccine targeting the X virus, and it has passed three phases of clinical trials, showing good effectiveness in preventing the X virus.” The number of likes, shares, and comments on the post was kept identical across conditions. Each post ended with the sentence: “To overcome the pandemic as soon as possible, get vaccinated now!,” calling on the public to actively receive the vaccine.

After reading assigned message, participants completed a questionnaire. They were asked to complete demographic questions (such as gender, age, education, and income), manipulation check items, attention check items, along with other items measuring vaccination intention and perceived credibility based on their genuine thoughts.

### 3.2 Participants

An a priori power analysis was conducted using the *pwr* package in R (64) to estimate a sample size needed for a medium effect size (*f* = 0.25). With an alpha of 0.05 and a power of 0.80, the projected minimum sample size needed for this effect was 180 participants. Using a simple random sampling technique, participants from China were recruited through Credamo, a professional online data collection platform. Credamo has a huge pool of participants (more than 3 million Chinese participants) with a demographic composition comparable to that of the Chinese population and coverage across all provinces in China. Unless otherwise stated, Credamo selects participants from its large and representative sample database using a simple random sampling method. The method of collecting data through Credamo has been widely recognized [e.g., (65–67)]. As compensation for their participation, each participant received 2 RMB. The data from participants who met any of the following criteria were excluded from the analysis: speeders (whose time to complete the survey was less than half of the median of the total survey duration, *n* = 21) (68); those who failed the attention check (*n* = 22) and those whose responses exhibited excessive uniformity (*n* = 17). After excluding unqualified participants, a total of 287 valid participants were retained. The final sample size exceeded the minimum requirement determined by the power analysis, thereby ensuring sufficient statistical power for the study.

### 3.3 Stimuli

In order to distinguish the different types of influencers, we used different influencer profile pictures and names, and informed the basic information of influencers before the experiment. Among them, the name of political influencer was “Guo Mingtao,” and his profile picture showed a man in suit. His identity information was described as a government official according to the following information: Guo Mingtao, male, a government official, responsible for disease prevention and control, health education, scientific research and public health policy support. As a Weibo blogger, he has 660,000 followers on his personal Weibo account and has a certain influence on the Internet. The expert influencer was named “Wang Qifan,” and his profile picture showed a man in a white coat. His identity information was described according to the following information: Wang Qifan, male, a respiratory specialist, has been engaged in the research and treatment of respiratory diseases for many years. Like Guo, he also has 660,000 followers on his personal Weibo account and has a certain influence on the Internet.

We also created two different message frames, and told the participants about either the benefits or the risks of the X-virus vaccine. The gain-framed message focused on the positive outcomes of getting vaccinated. For example, it stated that the vaccine could enhance immunity, protect one’s family, and help restore normal life. In contrast, the loss-framed message focused on the negative costs of not getting vaccinated. It warned that avoiding the vaccine could pose health risks, endanger one’s family, and prolong restrictions.

### 3.4 Measures

#### 3.4.1 Attention check

In order to screen out valid respondents, we included an attention check question from Stenlund et al. (69) in our questionnaire (e.g., “*To show that you are paying attention, please select disagree for this statement*.”). Participants who did not select this option would be excluded from the analyses.

#### 3.4.2 Manipulation check

To ensure the validity of the influencer type manipulation inspection tool, this study referred to the operational definitions of the concepts “political influencers” (7) and “expert influencers” (8). Two 7-point scale items have been designed for manipulatory checks, including: “*The poster is a government official*” and “*The poster is an expert in the field of respiratory diseases*.” Adapted from previous research (70), we designed the message framing manipulation with two similar statements. Participants rated their agreement with the items “*This post highlights the benefits of receiving the X-virus vaccine*” and “*This post highlights the risks of not receiving the X-virus vaccine*.” (All four manipulation-check items were measured on a 7-point scale, where 1 = Strongly disagree and 7 = Strongly agree.)

#### 3.4.3 Vaccination intention

We measured vaccination intention with five items adapted from previous research (71). Sample items included, “*I will get vaccinated against the X-virus within the next six months*” “*I intend to get vaccinated against the X-virus*” and “*I expect to get vaccinated against the X-virus*.” The scale was highly reliable (M = 5.80, SD = 0.89, *α* = 0.88).

#### 3.4.4 Perceived credibility

We measured perceived credibility using five items adapted from a previous study (34). Sample items included, “*I think the post published by the poster is credible*” “*I think the post published by the poster is trustworthy*” and “*I think the post is reliable*” This scale was also highly reliable (M = 5.70, SD = 0.85, *α* = 0.87).

### 3.5 Data analysis

Data analysis was performed using R 4.4.1 software. We first ran descriptive statistics to summarize participants’ demographic characteristics. Then we used independent-samples *t*-test to check whether the manipulations for influencer type and message framing worked as our intended. To address RQ1, we ran an ANOVA to examine the main effect of influencer types on vaccination intention. To test H1, we used Hayes’ PROCESS Macro Model 4 with 5,000 bootstrap iterations. In this model, influencer type serves as the independent variable, perceived credibility as mediating variables, and vaccination willingness as the dependent variable. An indirect effect was considered statistically significant if the 95% CI did not include zero. Meanwhile, to test H2, we conducted an ANOVA to examine the interaction between influencer type and message framing in predicting vaccination intention.

### 3.6 Ethical considerations

This study was approved by the School of Journalism and Communication at Henan University (Ethics Approval Number: 2025-115-001). Before the experiment began, we introduced the purpose and significance of the research to the participants and obtained informed consent.

## 4 Results

In this study, 120 participants were male, accounting for 41.81% of the total sample; 167 participants were female, accounting for 58.19% of the total sample. The average age of the participants was 30.20 years (SD = 7.64). 10 participants had a high school education or below (3.48%); 195 participants held a bachelor’s degree (67.94%); 78 participants held a master’s degree (27.18%); and 4 participants held a doctoral degree (1.40%). 8 participants reported an income below 1,000 RMB (2.79%); 33 participants earned between 1,000 and 3,000 RMB (11.50%); 32 participants earned between 3,001 and 5,000 RMB (11.15%); 71 participants earned between 5,001 and 8,000 RMB (24.74%); 56 participants earned between 8,001 and 10,000 RMB (19.51%); and 87 participants earned more than 10,001 RMB (30.31%). Their demographic information is presented in Table 1.

**Table 1:** Demographic information of participants (*N* = 287).

Characteristics	Category	Participants
Gender	Male	120 (41.81%)
	Female	167 (58.19%)
Age	Mean (SD)	30.20 (7.64)
Education	High school education or below	10 (3.48%)
	Bachelor’s degree	195 (67.94%)
	Master’s degree	78 (27.18%)
	Doctoral degree	4 (1.40%)
Income	Below 1,000 RMB	8 (2.79%)
	1,000 RMB − 3,000 RMB	33 (11.50%)
	3,001 RMB − 5,000 RMB	32 (11.15%)
	5,001 RMB − 8,000 RMB	71 (24.74%)
	8,001 RMB − 10,000 RMB	56 (19.51%)
	10,001 RMB or more	87 (30.31%)

We used the independent samples t-tests to examine the effectiveness of the manipulations for influencer type and message framing. The results showed that participants’ ratings for “The poster is a government official” were significantly higher than those of expert influencers under the condition of political influencers (*N* = 144, M = 6.60, SD = 0.82, SE = 0.07; *N* = 143, M = 1.28, SD = 0.55, SE = 0.05; *t* = 64.61, *p* < 0.001, Cohen’s *d* = 7.62); whereas the score for “The poster is an expert in the field of respiratory diseases” were significantly higher under the expert influencer condition than that of the political influencer (*N* = 144, M = 2.19, SD = 1.49, SE = 0.12; *N* = 143, M = 6.66, SD = 0.54, SE = 0.05; *t* = −33.85, *p* < 0.001, Cohen’s *d* = −3.99).

Under the gain frame condition, participants’ ratings for “This post highlights the benefits of vaccination” were significantly higher than those under the loss frame (*N* = 144, M = 2.37, SD = 1.53, SE = 0.13; *N* = 143, M = 6.39, SD = 0.68, SE = 0.06; *t* = −28.78, *p* < 0.001, Cohen’s *d* = −3.39); whereas under the loss frame condition, participants’scores for “This post highlights the risks of not getting vaccinated” were significantly higher than those under the gain frame (*N* = 144, M = 6.44, SD = 0.83, SE = 0.07; *N* = 143, M = 1.79, SD = 0.94, SE = 0.08; *t* = 44.52, *p* < 0.001, Cohen’s *d* = 5.26). The above results indicate that the manipulations of influencer type and message framing were successful.

To examine the effectiveness of random assignment, this study employed an analysis of variance (ANOVA) to test whether variables such as gender, age, education level, and income were randomly distributed across experimental groups. The results indicate that there were no significant differences among the groups in terms of gender [*F*(3, 283) = 0.48, *p* = 0.70], age [*F*(3, 283) = 0.28, *p* = 0.84], educational level [*F*(3, 283) = 0.84, *p* = 0.47], or income [*F*(3, 283) = 1.13, *p* = 0.34].

RQ1 aimed to explore whether there is a significant difference between types of influencers (political and expert) in persuading the public to get vaccinated. To address this question, ANOVA was employed to examine the main effect of influencer type on vaccination willingness. The results showed that the main effect of influencer type on vaccination intention was significant, *F*(1, 285) = 10.79, *p* < 0.01, *η*_p_^2^ = 0.04. Participants in the political influencer condition reported significantly higher vaccination intention (*N* = 144, M = 5.93, SD = 0.93, SE = 0.08) than those in the expert influencer condition (*N* = 143, M = 5.60, SD = 0.81, SE = 0.07). Thus, RQ1 was supported.

**Figure 1:**
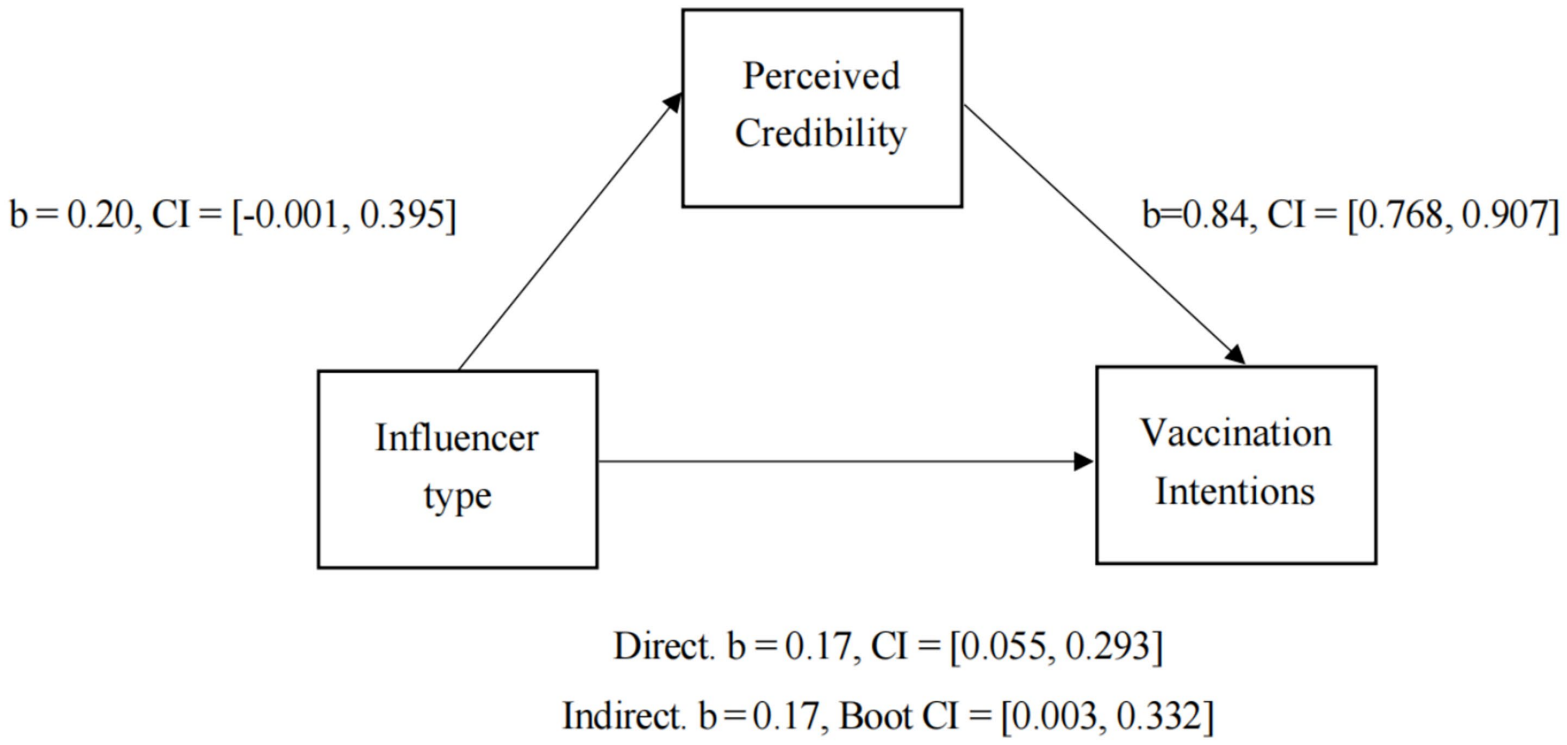
The mediation effect of perceived credibility between influencer type and vaccination intentions.

H1 posited that perceived credibility mediated the relationship between influencer type and vaccination willingness. We tested this mediation effect using PROCESS Macro Model 4 (72) in R. A bootstrap analysis based on 5,000 samples revealed that the mediating effect of perceived trust between influencer type and vaccination intention was significant. Specifically, regarding the indirect effect, *b* = 0.17, boot CI = [0.003, 0.332] (political influencers = 1, expert influencers = 0). The path diagram with coefficients is shown in Figure 1. H1 was supported.

**Figure 2:**
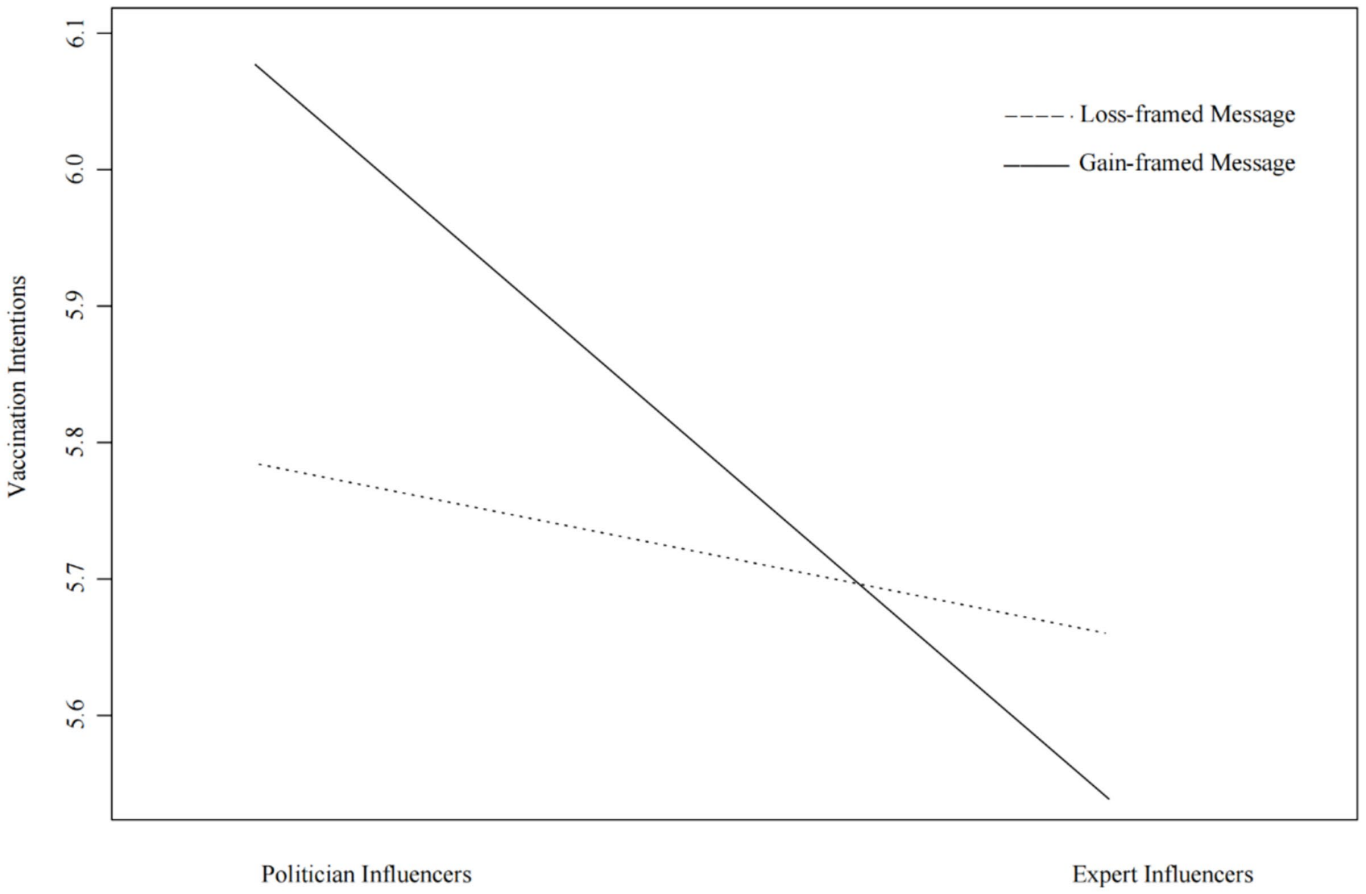
Interaction effects between influencer type and message framing on vaccination intentions.

H2 predicted that there was an interaction between influencer type and message framing in predicting vaccination intention. The results revealed a significant interaction between influencer type and message framing in influencing public vaccination intention [*F*(1, 283) = 4.248, *p* < 0.05, *η*_p_^2^ = 0.015]. When posts from political influencers were presented in a gain frame, they elicited higher vaccination intention compared to when presented in a loss frame. In contrast, when posts from expert influencers were presented in a gain frame, they were less effective in eliciting higher vaccination intention compared to when presented in a loss frame. Thus, H2 was supported (see Figure 2).

In summary, the findings indicate that there are significant differences in the persuasiveness of different types of influencers in public vaccination. Compared with expert influencers, political influencers were more persuasive in boosting public willingness to get vaccinated. Mediation analyses showed that the persuasive effect of influencers was indirectly achieved by improving the public’s perception of their credibility. Interaction analyses indicated that when political influencers used gain-framed messages, the public’s willingness to get vaccinated was higher than when they used loss-framed messages. On the contrary, when expert influencers used loss-framed messages, their persuasive effect was stronger than when they used gain-framed messages. Across all experimental conditions, the combination of political influencers and gain-framed messages elicited the highest willingness among the public to get vaccinated, while the combination of expert influencers and gain-framed messages resulted in the lowest.

## 5 Discussion

During the pandemic caused by an unknown virus in the future, it is particularly important to assess the effectiveness of different types of influencers in persuading the public to get vaccinated. This study explored how influencer types (political vs. expert) affect the public’s willingness to get vaccinated. The mediating role of perceived credibility between influencer type and vaccination willingness was also empirically tested. We further explored the interaction between influencer type and messages framing to reveal the corresponding matching effects. The results of this study can provide guidance for formulating more effective vaccine publicity strategies and enhancing the success of public health campaigns.

The results of this study show that political influencers are more effective in encouraging the public to get vaccinated than expert influencers. This result can be understood by the informational entropy-based notion of value. This theory says that information exists in small units called “grains,” and that the human brain has limited capacity to hold these grains. The entropy in the human mind (uncertainty or missing information) depends on how many information grains there are and how they are ranked in priority (73). This means that when the amount of information increases and there is no clear distinction or order of importance, informational entropy rises quickly, and the risk of losing or forgetting information also increases (73, 74). Compared with political influencers, expert influencers are usually linked to high-density professional information grains, such as vaccine efficacy data, clinical trial results, epidemiological models and so on. Because of cognitive inertia, the public is more likely to connect high informational entropy with expert influencers, which can lead to resistance to, or neglect of, the information they provide.

Mediation analysis indicated that perceived credibility mediated the relationship between influencer type on the willingness to get vaccinated. Participants found messages from political influencers to be more credible. This higher perceived credibility then increased their willingness to get the vaccine. This finding can be explained by the meaning transfer. Previous literature has shown that confidence in the government’s handling of a pandemic is an important predictor of public acceptance of vaccination (36). During the COVID-19 pandemic, through effective anti-pandemic measures, the public showed a high level of trust in the Chinese government (75). In China, the government enjoys relatively strong authority, and the public’s trust in politics is relatively stable. Due to the high alignment between political influencers and mainstream authorities, a “symbolic association” is formed between them. In this context, messages conveyed by political influencers are often perceived as representing the government’s stance. As a result, the public’s trust in the government shifts to political influencers, thus enhancing their influence during the pandemic. In addition, although vaccination involves public health issues, the decision-making process does not rely heavily on complex medical knowledge; on the contrary, it mainly depends on individuals’ perceptions of vaccine safety. Therefore, the expertise of expert influencers does not play an effective role in vaccine promotion, which in turn makes them less persuasive than political influencers. Certainly, the weaker persuasive effect of expert influencers may be related to the public’s message fatigue. Message fatigue is an aversive motivational state characterized by feelings of exhaustion and boredom resulting from prolonged and excessive exposure to similar and redundant information (76). During the COVID-19 pandemic, the public was constantly exposed to large volumes of repetitive messages from experts, leading to a sense of weariness and aversion toward expert sources.

Our analysis also revealed the significant interaction between influencer type and message framing on the willingness to be vaccinated. Specifically, gain-framed messages conveyed by political influencers were more effective than loss-framed messages. However, the opposite was true for expert influencers. When expert influencers conveyed information, loss-framed messages were more persuasive than gain-framed messages. We believe this happens because the influencer’s public image “matches” the tone of the message. Political influencers usually emphasize hope, collective interests, and social order. Gain-framed messages focus on positive outcomes such as protecting the family and returning to normal life, which is very suitable for this image. In contrast, expert influencers usually represent expertise. When expert influencers are paired with loss-framed messages, the public’s perception of potential risks is amplified, thus increasing their willingness to get vaccinated to avoid these risks.

## 6 Conclusion

During a pandemic caused by an unknown virus, persuading the public to get vaccinated is a key measure to end the pandemic as soon as possible. Through a 2 × 2 online experiment, this study examines the impact of influencer type on the public’s willingness to be vaccinated during the pandemic in the future. We found that political influencers were better than expert influencers at persuading people to get vaccinated. This effect mainly worked through higher perceived credibility among the public. We examine the matching effect between influencer type and message framing. Specifically, the gain frame (emphasizing the positive benefits of vaccination) amplifies the persuasive advantage of political influencers; the loss frame (emphasizing the negative consequences of non-vaccination) enhances the persuasive effect of expert influencers. This indicates that the public’s willingness to get vaccinated is not determined by a single factor, but is the result of the combined effect of influencer type and message framing. This research provides inspiration for information design in health communication, and also provides scientific theoretical guidance for the practice of vaccine promotion.

## 7 Theoretical, practical, and public policy implications

Theoretically, this study enriches the current understanding of the theoretical model of persuasion, especially in the context of the pandemic. The “match-amplification” effect found in the study breaks through the traditional single factor persuasion model (such as simple source effect or framing effect). It demonstrates that persuasiveness is the result of the joint effect of source characteristics and content characteristics. The study further finds that perceived credibility plays an important role in the influence of influencer type on the willingness to be vaccinated. This helps us understand the psychological mechanism behind the persuasion effect of influencers in the field of health communication. This study also provides an empirical basis for the application of the Bayesian Mindsponge Framework and the informational entropy-based notion of value in the field of public health. The research results show that the key to whether health information can be accepted is whether the transmitter has passed the audience’s “trust filter,” which is similar to the “information absorption-filter-integration” process emphasized in the Bayesian Mindsponge Framework. The different persuasion effects of different types of influencers in the field of health communication may be due to differences in the entropy (uncertainty) of the information they transmit in their daily lives.

This research has significant insights into the practice of vaccine promotion and policy formulation. A major finding is that political influencers are more persuasive than expert influencers in increasing the public’s willingness to get vaccinated. This highlights that political influencers, as important sources of information, play a crucial role in vaccine promotion during future pandemics. Governments can integrate different types of influencers into the public health communication system and encourage political influencers to take on greater social responsibility on matters such as vaccine promotion by formulating policies.

The study found that perceived credibility acted as a mediating role between influencer type and the willingness to be vaccinated. This finding reveals the potential mechanism of influencers’ persuasiveness, indicating that perceived credibility is a key factor affecting the effectiveness of persuasion. Therefore, when formulating persuasion strategies, we should pay more attention not only to influencers’ expertise and social influence, but also to their credibility among the public. Governments can also endorse influencers through official channels, so as to improve their perceived credibility among the public, thereby enhancing the effectiveness of vaccine information dissemination.

Further, the interaction between influencer type and message framing provides guidance for us. Strategies should match different influencers with appropriate message frames. Specifically, when vaccine-related information is delivered by political influencers, gain-framed messages should be adopted to promote vaccination. When it is delivered by expert influencers, loss-framed messages are likely to be more effective.

## 8 Limitations and opportunities for future research

This research has certain limitations. It is worth noting that this study fictionalized a pandemic similar to COVID-19 as the background of the experiment. Participants were informed before the experiment that the experimental scenario and vaccine information were fictional, so the individual’s reaction may be different from the real scenario. Therefore, future research can further explore the moderating effect of perceived authenticity in the process of persuasion. This study focused on participants’ vaccination intentions and did not investigate the relationship between these intentions and actual vaccination behavior. Although behavioral intentions can generally predict actual behavior, self-reported intentions may be influenced by social desirability bias. Thus, future research should further examine actual vaccination behavior. This study only verifies the interaction between influencer type and message framing. Future research can further consider the interaction between influencer type and other information characteristics (for example, information appeals, information evidence). Furthermore, while the study distinguished between political influencers and expert influencers, it did not directly measure the extent to which participants perceived different types of influencers as government representatives. Future research can incorporate relevant variables, including political ideology, trust in government, prior vaccine attitudes, health literacy to reveal the potential psychological mechanisms through which influencer types affect public vaccination intention. This study chose Weibo as a social media platform. The sample comes from Chinese people and has certain cultural characteristics. The research results are not applicable to other countries or cultural backgrounds. In the United States, for example, strong political polarization may shape how people respond to political figures, and reactions may depend on party identity and political views. Comparative studies across countries and cultures could show how the persuasive effects of political and expert influencers vary in different vaccine campaigns.

## Data Availability

The original contributions presented in the study are included in the article/supplementary material, further inquiries can be directed to the corresponding author.
